# Mammographic breast density in infertile and parous women

**DOI:** 10.1186/s12905-016-0284-8

**Published:** 2016-02-09

**Authors:** Meggiorini Maria Letizia, Vestri Anna Rita, De Stefano Maria Grazia, Cipolla Valentina, Bellati Filippo, Maffucci Diana, Nusiner Maria Paola, Aragona Cesare, De Felice Carlo

**Affiliations:** 1 Department of Gynecologic-Obstetrical and Urologic Sciences, “Sapienza” University of Rome, Viale del Policlinico, 155, Rome, 00161 Italy; 2 Department of Radiological Sciences, “Sapienza” University of Rome, Viale del Policlinico, 155, Rome, 00161 Italy; 3 Department of Public Health and Infectious Diseases, “Sapienza” University of Rome, Viale del Policlinico, 155, Rome, 00161 Italy

**Keywords:** Breast density, Infertility, Parous women

## Abstract

**Background:**

Mammographic breast density is a useful marker for breast cancer risk, as breast density is considered one of the strongest breast cancer risk factors. The study objective was to evaluate and compare mammographic breast density in infertile and parous women, as infertility may be associated with high breast density and cancer occurrence.

**Methods:**

This study evaluated mammographic breast density using two different systems, BIRADS and Boyd. A selected patient population of 151 women with primary infertility (case group) was compared to 154 parous women who had at least one previous pregnancy (control group). Both groups were premenopausal women aged ≥35.

**Results:**

Evaluation of mammographic features showed that 66.9 % of case group patients and 53.9 % of control group patients were classified BIRADS-3/BIRADS-4; *p* < 0.05. Adjusted Odds ratio for the case group in the categories BIRADS-3/BIRADS-4 was 1.78 (95 % CI: 1.10-2.89). Using the Boyd classification system, 53.6 % of case group patients and 31.8 % of control group patients were classified E/F; *p* < 0.05. Adjusted Odds ratio for case group patients in Boyd categories E/F was 2.05 (95 % CI: 1.07-3.93).

**Conclusions:**

Both systems yielded a higher percentage of increased breast density in the case group. Boyd and BIRADS classification systems indicate to what extend breast cancer lesions may be missed on mammography due to masking by dense tissue. Therefore, patients with a high BIRADS or Boyd score should undergo further investigation.

## Background

Infertility is defined as the failure to conceive after one year of regular unprotected intercourse, and prevalence is approximately 10 %-15 % in Western countries [1]. The demand for infertility services is steadily increasing, and so is the average age of women undergoing in vitro fertilization (IVF) [2, 3]. Infertility treatments implying medication and interventional procedures may modify the hormonal environment and be cofactors in cellular changes leading to cancer development [4, 5]. However, few studies are reported on breast cancer risk in infertile women who have undergone IVF treatment [6–8].

The association between breast cancer and infertility has not been consistently studied, but it may be explained by hormonal disorders linked to infertility, a different reproductive risk factor profile in infertile women, or by a combination of these two factors.

Two decades ago, Sellers et al. reported a significantly higher risk of developing breast cancer in nulliparous married women who tried and failed to become pregnant [9]. Weiss et al. [10] found that among women with a first full-term delivery at the age of 35 or older, fertility problems were associated with a two-fold increased risk of breast cancer. In contrast, Venn et al. [5] and Klip et al. [8] summarized the association between infertility and breast cancer and concluded that infertile women did not appear to be at higher risk for breast cancer. However, the possible influence of infertility itself on the risk of breast cancer occurrence should be taken into consideration [11].

The Italian National Federation for Breast Cancer (FONCAM) guidelines 2005 recommend mammography starting at 35 years in women who undergo hormone therapy [12].

At the Breast Unit of our institution (Policlinico Umberto I, Rome), all women undergoing fertility treatment are submitted to breast examination, and mammography is performed according to the FONCAM recommendations for women aged ≥35.

Mammographic breast density reflects the relative proportion of glandular and stromal tissue to fatty tissue in the breast. High breast density has been strongly associated with increased risk of breast cancer [13–15], and this parameter could thus be a useful marker for breast cancer risk and be considered one of the strongest risk factors for breast cancer [15]. Breast density is often associated with reproductive and menstrual factors including serum estrogen and progesterone concentrations [16]. Furthermore, breast density is inversely correlated with the accuracy of mammography and therefore a measurement of density conveys information on the difficulty of detecting cancer in a mammogram.

In a previous study, we found that 68 % of women with primary infertility had dense breast tissue according to the BIRADS score compared with 37 % of premenopausal women in a general population [17].

In the present study, two different systems, BIRADS and Boyd, were employed to evaluate mammographic breast density in two selected groups: a case group consisting of infertile women and a control group consisting of parous women. The results obtained in the two groups were subsequently compared.

## Methods

This study was carried out from January 2007 to November 2009. Data collection methods and study design were approved by the institutional review board of the University of Rome “Sapienza” and all patients signed an informed consent form. The study included women with primary infertility referred to our Breast Unit at the Department of Gynaecology and Obstetrics, University of Rome “Sapienza” as well as a control group selected from the database of the Department of Radiology.

A total of 440 patients were referred to our Breast Unit from the IVF Centre for a clinical breast examination before undergoing specific infertility treatment. Of these patients, 51 % were >35 years old, 37 % had previously undergone fertility drug treatment and 9 % had secondary infertility. Of the 440 patients, 151 (34 %) were women aged >35 with primary infertility who had never undergone fertility drug treatment (case group). The case group was compared to a control group consisting of 154 parous women selected from the database of the Department of Radiology who had at least one previous pregnancy (average 1.3 children per woman). The control group patients had undergone mammography examination for other reasons, such as family history or clinical suspicion of breast tumours, and they were negative for cancer. The age range of the control group was chosen in accordance with the case group: premenopausal women age ≥35. The information collected in both groups included: age, family history of breast cancer with at least 2 first degree relatives, previous administration of hormonal contraceptive therapy (HCT), age at menarche, previous pregnancy or etiology of infertility (if known) as well as height and weight for the calculation of body mass index (BMI). According to the FONCAM recommendations, all recruited case group patients underwent clinical examination and X-ray mammography (XRM) after signing the informed consent form. In all cases, conventional XRM was performed at our Department of Radiological Science using digital image formation and computed radiography. At least two views per breast were obtained.

All mammograms were classified using BIRADS and Boyd scales. Classification was carried out by three physicians (two radiologists and a breast specialist) blinded to the clinical data in accordance with the guidelines of the American College of Radiology (ACR) BIRADS. Diagnostic quality of the mammograms was assessed according to the PGMI evaluation system that includes four image quality categories: P (perfect), G (good), M (moderate) and I (inadequate).

BIRADS is a qualitative system, but the recently modified, more quantitative version was employed [18].

According to the BIRADS lexicon, patients were assigned to one of the four categories of breast parenchymal density distribution: BIRADS-1: the breast is almost entirely fat (glandular parenchyma <25 % of the total area of both breasts); BIRADS-2: scattered fibroglandular densities (25 %-50 %); BIRADS-3: heterogeneously dense breast tissue (51 %-75 %); BIRADS-4: extremely dense (>75 % glandular tissue). The Boyd scale is based on a visual estimation of the proportion of mammographic density in the projected area, divided into six categories of unequal intervals: type A: 0 %; type B: 0-10 %; type C:10-25 %; type D: 25-50 %; type E: 50-75 % and type F: >75 %. In case of contradictory judgments, the classification assigned by at least two readers out of three was considered correct.

Patients were excluded from the study if mammography revealed focal disease, and/or if further diagnostic tests such as breast ultrasound, breast magnetic resonance or needle biopsy were required. To assess whether classification as Dense Breast (DB) and Non Dense Breast (NDB) was consistent, agreement between the three referents was tested using Cohen’s kappa before further statistical analysis. It was decided to divide BIRADS into two categories and BOYD into three categories because absolute frequencies for certain categories were very small and this would create problems in the multivariate analysis.

The sample of 151 women in the case group and 154 in the control group were evaluated as having a 80 % power to detect an OR (Odds Ratio) of 2 at a 0.05 significance level for an exposure (dense breast according to BIRADS score) expected the control group equal to 40 %. The proportional odds were tested using the Brant test. For both groups of cases, the proportional odds assumption was verified (chi square = 2.316 *p* = 0.678 in the case group; chi square = 4.106 *p* = 0.392 in the control group).

Continuous variables were presented as mean ± Standard Deviation (SD), and differences were evaluated by the Student’s *t* test or the Mann–Whitney test. Categorical variables were expressed as count and percentages. To assess the association the chi-square test or Fisher’s exact test were used as appropriate. Multivariate analyses (ordered logistic regression and binary logistic regression) were used to calculate ORs, including interacting or confounding effects of age, BMI, family history and age at menarche. All tests were two-tailed, and *p*-value <0.05 was considered significant. All computations were carried out using STATA v.12.

## Results

Mean age of the 151 case group patients was 38.96 ± 2.88 and mean BMI was 23.15 ± 3.88. All were affected by primary infertility: 57.8 % sine causa, 37 % ovulatory factors and 5.2 % endometriosis. Of this group, 6.6 % had a positive family history of breast cancer and 40.4 % had received hormonal contraceptive therapy (HCT). Mean age at menarche was 12.34 ± 1.49.

Mean age of the 154 control group patients was 41.79 ± 3.09 and mean BMI was 22.58 ± 3.05. Of this group 52 % had a positive family history of breast cancer and 44.2 % had received contraceptive therapy. Mean age at menarche was 12.44 ± 1.47.

The mean age difference between the two groups was statistically significant, but difference in BMI values was not statistically significant (Table 1).

**Table 1:** Characteristics of the two study groups

	Case group (151 women)	Control group (154 women)	*p*
BMI (kg/m2) (mean ± SD)	23.15 ± 3.88	22.58 ± 3.05	0.591
Age (mean ± SD)	38.96 ± 2.88	41.79 ± 3.09	<0.001
Family history of Breast Cancer (%) No.	(6.6 %) 10	(52 %) 80	<0.0001
Previous use of HCT (%) No.	(40.4 %) 61	(44.2 %) 68	0.308
Age at menarche (mean ± SD)	12.34 ± 1.49	12.44 ± 1.47	0.148

All mammograms included in the study were classified as P (perfect) or G (good) according to the PGMI evaluation system.

Evaluation of mammographic features showed that 33.1 % (50/151) of case group patients and 46.1 % (71/154) of control group patients were classified BIRADS-1/BIRADS-2; *p* < 0.05, and that 66.9 % (101/151) of case group patients and 53.9 % (83/154) of control group patients were classified BIRADS-3/BIRADS-4; *p* < 0.05 (Table 2). Adjusted OR for the case group vs. the control group in categories BIRADS-3/BIRADS-4 was 1.78 (95 % CI: 1.10-2.89); *p* = 0.02 (Table 3).

**Table 2:** Evaluation of mammographic features using the BIRADS and Boyd systems

	Case group (151 women)	Control group (154 women)	*p*
BIRADS-1/BIRADS-2	33.1 %	46.1 %	0.026
BIRADS-3/BIRADS-4	66.9 %	53.9 %	0.026
Boyd Cat. A/B	19.2 %	22.7 %	NS
Boyd Cat. C/D	27.2 %	45.5 %	<0.0001
Boyd Cat. E/F	53.6 %	31.8 %	<0.0001

**Table 3:** Odds ratios, 95 % confidence intervals and *p*-values for higher BIRADS categories (BIRADS 3/4) for characteristics of the study population (Odds ratio for each variable is adjusted for the remaining variables shown in the table)

BIRADS Cat. 1/2	OR	Confidence interval 95 % lower bound upper bound		*p* value
Family history	1.32	0.59	2.95	0.50
Age at menarche	0.86	0.69	1.06	0.15
BMI normal	1.52	0.46	5.03	0.49
BMI overweight	0.50	0.13	1.86	0.30
BMI obese	0.45	0.10	2.06	0.31
Age 35-39	3.99	1.26	12.61	0.02
Age ≥40	2.46	0.77	7.91	0.13
BIRADS 3/4	1.78	1.10	2.89	0.02

Boyd classification of mammographic features showed that 19.2 % (29/151) of case group patients and 22.7 % (35/154) of control group patients were assigned to categories A and B (*p* > 0.05); 27.2 % (41/151) of case group patients and 45.5 % (70/154) of control group patients were assigned to categories C and D (*p* < 0.05); 53.6 % (81/151) of case group patients and 31.8 % (49/154) of control group patients were assigned to categories E and F; (*p* < 0.05) (Table 2).

Adjusted OR for the case group vs. the control group in Boyd categories E and F was 2.05 (95 % CI: 1.07-3.93) (*p* = 0.03). Adjusted OR for categories C and D vs. categories A and B was 0.75 (95 % CI:0.39-1.46) (*p* = 0.39) (Table 4).

**Table 4:** Odds ratios, 95 % confidence intervals and *P* values for higher Boyd grades for characteristics of the study population (Odds ratio for each variable is adjusted for the remaining variables shown in the table)

Boyd Classification Cat. A/B	OR	Confidence interval 95 % lower bound upper bound		*P* value
Family history	1.06	0.46	2.47	0.89
Age at menarche	0.96	0.79	1.18	0.72
BMI normal	1.42	0.42	4.98	0.59
BMI overweight	0.56	0.12	1.98	0.34
BMI obese	0.45	0.09	2.45	0.32
Age 35-39	3.13	1.12	9.77	0.04
Age ≥ 40	1.45	0.83	2.52	0.20
Boyd Cat. C/D	0.75	0.39	1.46	0.39
Boyd Cat. E/F	2.05	1.07	3.93	0.03

## Discussion

Breast cancer is the most common malignancy in women and a leading cause of cancer-related death worldwide [15, 19]. In the absence of a known preventable cause of breast cancer, the single most important factor in reducing mortality is early detection. Mammography and ultrasound are currently the techniques of choice for breast cancer evaluation. The sensitivity of mammography varies and is influenced by breast density. Dense fibroglandular tissue is the most important inherent limitation of mammography in the diagnosis of breast cancer [12], and dense breast tissue is considered a risk factor in the subsequent development of breast cancer [19].

Mammographic breast density is very often linked to cancer due to masking bias, as malignant lesions have the same x-ray attenuation properties as fibroglandular tissue [19, 20]. In our previous study, we analysed a selected population of women with primary infertility and we found that 68 % of patients had dense breasts according to the BIRADS score [17]. The study in question presented some limitations: it lacked a control group and used only the BIRADS system, which was originally a morphological assessment.

This is the first study that has investigated breast density of infertile women referred to assisted conception services and compared the outcome with a group of premenopausal parous women aged ≥35. In this study, two methods were used to assess mammographic density, BIRADS and Boyd, and both confirmed previous results for the infertile women. The percentage of dense breasts in the group of women with primary infertility was higher using both BIRADS (66.9 % vs. 53.9 %, *p* < 0.05) and Boyd (53.6 % vs. 31.8 %, *p* < 0.05) compared to parous woman. One of the most important limitations of this study is the difference in the mean age between the two groups. Mean age of the control group was higher, because mammographic screening and other preventive measures before age 40 are not recommended except in women at high risk of breast cancer [12]. According to the FONCAM guidelines, the first diagnostic approach recommended before age 40 is clinical examination. If suspicious signs for malignancy are found, ultrasound is performed, followed by a mammogram if necessary [12]. Therefore, from the database of the Department of Radiology, we selected women who had undergone mammography examinations for various reasons. This caused a slight difference between the mean age in the two study groups. Sensitivity and specificity of mammographic evaluation is lower in younger women than in older women because younger woman more often have dense breast tissue [21]. Therefore, we used multivariate analysis to take into account potential confounding factors, such as age, BMI and family history. Nevertheless, dense breast tissue was more frequent in the case group as established according to the Boyd and BIRADS systems. The role of nulliparity as a risk factor for breast density has been discussed in several studies [13, 22]. There might be a biological relationship between parity and the risk of breast cancer given that high breast density is present in most nulliparous women [10, 14, 17]. Furthermore, nulliparous women often present a large quantity of undifferentiated epithelial breast tissue, which is more susceptible to carcinogenetic stimuli such as endogenous and exogenous female hormones [23].

## Conclusions

In conclusion, this study confirms that patients with primary infertility have denser breast tissue and may be at risk for breast cancer.

The Boyd and BIRADS systems were not developed to quantify the risk, but to allow an interpreting radiologist to indicate the level of concern that a cancer lesion in the breast might be missed on mammography due to masking by dense tissue.

Infertile women with high BIRADS or Boyd scores, who undertake IVF treatment, should be investigated with appropriate diagnostic tools including magnetic resonance imaging (MRI) to reduce masking bias.

## Abbreviations

BIRADS: Breast imaging-reporting and data system
IVF: In vitro fertilization
F.O.N.Ca.M.: Forza operativa nazionale sul carcinoma mammario
HCT: Hormonal contraceptive therapy
BMI: Body mass Index
XRM: X-Ray mammography
ACR: American college of radiology
DB: Dense breast
NDB: Non dense breast
OR: Odds ratio
